# Retrospective study of pregnant women placed under expectant management for persistent hemorrhage

**DOI:** 10.1007/s00404-013-2972-z

**Published:** 2013-07-26

**Authors:** Shigeru Aoki, Megumi Inagaki, Kentaro Kurasawa, Mika Okuda, Tsuneo Takahashi, Fumiki Hirahara

**Affiliations:** 1 Perinatal Center for Maternity and Neonate, Yokohama City University Medical Center, 4-57 Urafunecyou, Minami-ku, Yokahama, Kanagawa 232-0024 Japan; 2 Department of Obstetrics and Gynecology, Yokohama City University Hospital, Yokohama, Japan

**Keywords:** Persistent subchorionic hematoma, Chronic abruption, Persistent hemorrhage, Expectant management

## Abstract

**Objective:**

To examine the clinicopathological characteristics of pregnant women who presented with intermittent hemorrhage occurring throughout pregnancy until delivery.

**Method:**

A total of 24 women with intermittent hemorrhage occurring throughout pregnancy were categorized into two groups, a group with persistent subchorionic hematoma (PSH) and another with chronic abruption (CA), and the pregnancy outcomes were compared between the two groups. The data were expressed as medians (range).

**Results:**

There were 18 women in the PSH group and 6 women in the CA group. The median gestational age at delivery was 27.9 (22.1–33.4) weeks in the PSH group and 32.9 (24.3–33.1) weeks in the CA group, revealing a significantly earlier gestational age at delivery in the former group (*p* = 0.014). The percentage of the women developing acute abruption tended to be higher in the CA group [66.7 % (4/6)] than in the PSH group [26.3 % (5/18)]. Small for gestational age (SGA) infants and neonatal chronic lung disease were seen at high incidences, but only in the PSH group [21.1 % (4/18) and 42.1 % (8/18), respectively].

**Conclusion:**

PSH was associated with earlier gestational age at delivery, a higher incidence of SGA infants, and poorer pregnancy outcomes than CA.

## Introduction

Pregnant women are occasionally encountered in clinical settings, in whom hemorrhage occurs intermittently throughout pregnancy until delivery and abruptio placentae is suspected, but no abnormalities are detected on fetal heart rate monitoring making both diagnosis and treatment difficult. This condition appears to be most often attributable to chronic abruption, a concept proposed by Naftolin et al. [1]. However, many points about the pathologic entity of chronic abruption still remain unclear, and no consensus has been reached regarding the treatment policy.

Herein, we examined 24 pregnant women in whom hemorrhage occurred intermittently throughout pregnancy until delivery by broadly categorizing them as the group in which subchorionic hematoma was present from the first trimester and episodes of hemorrhage occurred intermittently until delivery, or as the group in which peripheral placental separation occurring in the second to third trimester could be managed expectantly without impairing the well-being of the fetus or the health of the mother. Since not only the timing of onset of hemorrhage, but also the gestational age at delivery and the pregnancy outcome differed significantly between the two groups, we retrospectively compared the clinical features of these groups. We also examined whether or not appropriate tocolysis could be achieved with a tocolytic agent.

## Materials and methods

From among 11,766 women who delivered during the period between January 2000 and June 2012 at the Perinatal Center for Maternity and Neonate, Yokohama City University Medical Center, Yokohama, Japan, the data of women in whom hemorrhage occurred intermittently throughout pregnancy until delivery, but could be managed expectantly, were retrospectively examined.

Pregnant women who met the following criteria were defined as having persistent hemorrhage requiring expectant management: (1) intermittent hemorrhage persisting during pregnancy until delivery; (2) placenta previa excluded; (3) exclusion of cervical disease as the source of bleeding; (4) expectant management possible; (5) presence of macroscopic retroplacental hematoma detected at delivery; and (6) delivery at 22 weeks of gestation or later.

Twenty-four women met the above criteria, and were broadly categorized as the group in which subchorionic hematoma was present from the first trimester and intermittent hemorrhage occurred until delivery [persistent subchorionic hematoma (PSH) group, *n* = 18] or the group in which peripheral placental separation that occurred in the second to third trimester could be managed expectantly without impairing maternal and fetal well-being [chronic abruption (CA) group, *n* = 6].

As a policy for expectant management, pregnant women in whom hemorrhage occurred in the second trimester or later were hospitalized for management as a general rule, and underwent ultrasound examination at least twice a week. For the women who were hospitalized at 22 weeks of gestation or later, regular blood tests, including at least once weekly coagulation tests, were carried out in addition to the fetal heart rate monitoring performed at least once daily. In both groups, uterine contractions accompanied by unpleasant subjective symptoms were managed by bed rest. On the basis of the physical findings, leucocyte/CRP values, intravenous tocolytic and/or antibiotic therapy was administered, on a case-by-case basis, at the attending gynecologist’s discretion. For all women who were between 23 weeks ± 0 day and 33 weeks ± 6 days of gestation in whom delivery was expected within 1 week, betamethasone was administered to facilitate fetal lung maturation. The background characteristics of the women, including the age, parity, multiplets, use of anticoagulant drugs, and gestation by in vitro fertilization, and the main outcomes, including the gestational age at delivery, timing of onset of the hemorrhage, need for tocolysis, occurrence of premature rupture of the membranes (PROM), complicating oligohydramnios, the maternal condition that developed acute abruption, and prevalence of chronic lung disease in the neonates, were compared between the two groups. Oligohydramnios is defined as a condition in which the amniotic fluid index is <5 cm or the amniotic fluid pocket is <2 cm, and acute abruption is defined as a condition of severe hemorrhage for which forced delivery is deemed necessary or a state in which the amount of hemorrhage is small, but typical abruptio placentae is diagnosed on the basis of uterine tenderness or back pain, high-frequency contractions, and late deceleration on fetal heart rate monitoring, and forced delivery is deemed necessary. These diagnoses were definitely made when a fresh retroplacental hematoma was identified by the post-delivery examination of the placenta. PROM was diagnosed when the result of the test for detection of the human insulin-like growth factor binding protein-1 (IGFBP-1) was positive.

The data are represented as medians (range) or frequencies (percentage). Microsoft Excel and SPSS were used for the statistical analyses. We applied the Mann–Whitney *U* test for analyzing continuous variables. Fisher’s exact tests were used to detect differences in categorical data by group. The level of statistical significance was set at *p* < 0.05.

## Results

No significant differences were found in the background characteristics between the two groups (Table 1). Hemorrhage started at a median gestation of 10.0 weeks (5.0–18.5 weeks) in the PSH group and at a median gestation of 29.2 weeks (23.7–33.0 weeks) in the CA group. In both groups, unpleasant uterine contractions accompanied by subjective symptoms were often seen in addition to hemorrhage, and intravenous tocolytic agents were administered in 88.9 % of women (16/18) in the PSH group and in all women (6/6) in the CA group. While PROM was seen in most women (17/18) in the PSH group, it was seen in 2 of the 6 women in the CA group (*p* = 0.006). Membrane rupture in all cases was difficult to differentiate due to hemorrhage, and diagnosis was made using reagents for detecting IGFBP-1. In most cases, membrane rupture was diagnosed on the basis of a positive response on the test rather than by macroscopic detection of obvious outflow of amniotic fluid. Premature chemical rupture of the membranes is defined as a state in which no amnion rupture is observed, but the permeability of the amniotic membrane is enhanced and the result of the highly sensitive rupture of membrane test, such as that involving the use of the IGFBP-1 reagent, is positive [2]. In our subjects, the possibility of a pseudo-positive response due to the premature chemical rupture of the membranes cannot be ruled out, although pigment instillation into the amniotic cavity is not performed at our hospital.

**Table 1:** Background characteristics

	PSH group (*n* = 18)	CA group (*n* = 6)	*p* value
Age	30 (24–37)	33 (22–39)	0.203
Primipara	9/18 (50.0 %)	3/6 (50.0 %)	1.000
Multiplets	0/18	0/6	–
Use of anticoagulant agents	0/18	0/6	–
In vitro fertilization pregnancy	1/18 (5.5 %)	0/6	1.000

Values are presented as medians (range) or frequencies (percentages)

*PSH group* persistent subchorionic hematoma group, *CA group* chronic abruption group

Oligohydramnios was seen only in the PSH group (8/18, 44.4 %), and in none of the women of the CA group (0/6, 0 %); however, no macroscopic outflow of amniotic fluid was observed, and the effect of factors other than PROM was suspected.

The hematoma was detected by ultrasonography immediately after the occurrence of hemorrhage in all cases of the PSH group, although the ultrasonographic detection became increasingly difficult with advancing the gestational age and immediately before delivery, the hematoma could be identified by ultrasonography in only 9 of the 18 women (50.0 %) in this group. On the other hand, the hematoma could be ultrasonographically identified immediately before the delivery in 4 of the 6 women (66.7 %) of the CA group.

The median gestational age at delivery was 27.1 weeks (22.1–35.4 weeks) in the PSH group and 33.2 weeks (26.4–35.1 weeks) in the CA group, revealing a significantly earlier gestational age at delivery in the former group (*p* = 0.017). The cesarean section rate was 61.1 % (11/18) in the PSH group and 66.7 % (4/6) in the CA group, revealing no significant difference between the two groups. However, the percentage of the women developing acute abruption tended to be higher in the CA group [66.7 % (4/6)] than in the PSH group [22.2 % (4/18)] (*p* = 0.129). In the CA group, the indication for cesarean section was abruptio placentae in all cases, while cesarean section was indicated for non-reassuring fetal status (NRFS) in five, abruptio placentae in four, and others in two women of the PSH group; thus, NRFS was the most common reason for cesarean section.

None of the women in either group exhibited any abnormalities in the coagulation tests performed periodically during the period from admission to delivery, or showed disseminated intravascular coagulation.

Macroscopic examination of the placenta after delivery identified placenta extrachorialis in a high percentage of women in both the PSH group (7/18, 38.8 %) and the CA group (3/6, 50.0 %).

The median birth weight was 738 g (436–2,464 g) in the PSH group and 1,833 g (746–2,696 g) in the CA group, showing the difference depending on the gestational age at birth. Meanwhile, the median umbilical artery blood pH at delivery was 7.312 (6.916–7.344) in the PSH group and 7.317 (7.125–7.422) in the CA group, being almost the same in the two groups. Infants who were small for gestational age and neonatal chronic lung disease were seen only in the PSH group [3/18 (16.6 %) and 8/18 (44.4 %), respectively], and there were no cases in the CA group.

Two neonatal deaths occurred after delivery at 22 weeks of gestation in the PSH group; both deaths were caused by immaturity of the infants due to the premature delivery (Table 2).

**Table 2:** Main outcomes

	PSH group (*n* = 18)	CA group (*n* = 6)	*p* value
Time of onset of the hemorrhage (weeks)	10.0 (5.0–18.5)	29.2 (23.7–33.0)	<0.001*
Use of tocolytic agents	16/18 (88.9 %)	6/6 (100 %)	1.000
Premature rupture of membranes	17/18 (94.4 %)	2/6 (33.3 %)	0.006
Oligohydramnios	8/18 (44.4 %)	0/6 (0 %)	0.066
Ultrasound detection of hematoma	9/18 (50.0 %)	4/6 (66.7 %)	0.649
Gestational age at delivery (weeks)	27.1 (22.1–35.4)	33.2 (26.4–35.1)	0.017*
Interval between onset of the hemorrhage and delivery (days)	103 (58–192)	18.5 (3–64)	<0.001*
Cesarean section	11/18 (61.1 %)	4/6 (66.7 %)	1.000
Developing acute abruption	4/18 (22.2 %)	4/6 (66.7 %)	0.129
Placenta extrachorialis	7/18 (38.8 %)	3/6 (50 %)	0.665
Birth weight (g)	738 (436–2,464)	1,883 (746–2,696)	0.014*
Umbilical artery pH at delivery	7.312 (6.916–7.344)	7.317 (7.125–7.422)	0.680
Small-for-gestational-age infants	3/18 (16.6 %)	0/6 (0 %)	0.546
Neonatal chronic lung disease	8/18 (44.4 %)	0/6 (0 %)	0.066
Neonatal death	2/18 (11.1 %)	0/6 (0 %)	1.000

Values are presented as medians (range) or frequencies (percentages)

* Statistically significant

*PSH group* persistent subchorionic hematoma group, *CA group* chronic abruption group

## Discussion

Pregnant women presenting with intermittent hemorrhage that occurred throughout pregnancy until delivery were categorized into two groups (the PSH and CA groups) according to the timing of start of the hemorrhage, and the characteristics and outcomes of the two groups were compared. As compared with the CA group, the PSH group was characterized by earlier gestational age at delivery, a higher incidence of neonatal chronic lung disease, and poorer outcomes. Chronic abruption is a condition in which hemorrhage associated with retroplacental hematoma formation is arrested [3]. In the so-called acute abruption, placental abruption rapidly occurs as a result of decidual spiral artery rupture, while chronic abruption is caused by venous hemorrhage occurring in the area surrounding the placenta; thus, a retroplacental hematoma is formed, with external hemorrhage as the main sign, without progression to placental abruption [4, 5].

Clinical diagnosis of a placental hematoma is difficult, and clear criteria do not exist to define the term “chronic abruption”. Many reports describe chronic abruption as a condition in which a subchorionic hematoma that forms in the first trimester does not disappear and hemorrhage continues even into the second and third trimesters of pregnancy [6, 7].

In other words, while both persistent subchorionic hematoma and chronic abruption may essentially represent peripheral placental hemorrhage [8], as the results of this study suggest, the pregnancy outcome differed significantly between the two groups.

Elliott et al. [7] classified the women meeting the following criteria as showing the chronic abruption-oligohydramnios sequence (CAOS), and examined 24 pregnant women who satisfied the criteria: (1) clinically significant vaginal bleeding persisting for at least 7 days without placenta previa or uterine cervical lesions, (2) amniotic fluid volume initially documented as normal, and (3) oligohydramnios (amniotic fluid index <5 cm) eventually developing without concurrent evidence of ruptured membranes. The results of this analysis revealed that the gestational age at delivery was earlier (26.1 vs. 31.0 weeks) and the incidence of PROM was higher (71 vs. 50 %) in the 14 women who began to have episodes of hemorrhage at 20 weeks of gestation or earlier than in the 10 women who had the first hemorrhage after 20 weeks of gestation. One possible cause of the PROM is that the bleeding dissects the amnion and chorion away from their decidual attachments, thereby impairing blood flow and inducing membrane rupture [7]. In their study, the oligohydramnios was detected in about 60 % (24/40) of women with chronic abruption. Such oligohydramnios has been suggested to be attributable to inadequate placental function leading to inadequate fetal renal perfusion [9].

The median gestational age was 27.1 weeks (22.1–35.4 weeks) at delivery in the PSH group, with incidences of PROM and oligohydramnios being 94.4 % (17/18) and 44.4 % (8/18), respectively. This may suggest that peripheral placental abruption occurred even in the PSH group, leading to chronic placental dysfunction that finally resulted in oligohydramnios, and NRFS associated with the placental dysfunction.

Moreover, the high incidence of neonatal chronic lung disease in the PSH group may be explained not only by the difference in the gestational age at delivery between the two groups, but also by the effect of the hemorrhage persisting over an even more prolonged period in this group, which would have induced diffuse chorioamniotic hemosiderosis and resulted in the uptake of hemosiderin in the amniotic fluid. Diffuse chorioamniotic hemosiderosis is a pathological finding of the placenta indicating the deposition of hemosiderin within the chorionic plate or membrane, consequent to the phagocytosis of hemoglobin or hemoglobin breakdown products by macrophages [9]. It is believed to be induced by chronic placental hemorrhage, and is speculated to lead to fetal lung injury when the fetus aspirates the amniotic fluid contaminated with blood [10]. The higher incidence of neonatal chronic lung disease in the PSH group, which is characterized by hemorrhage persisting over a more prolonged period than that in the CA group, does not contradict the hypothesis that diffuse chorioamniotic hemosiderosis is the cause of the neonatal chronic lung disease.

There have been some previous reports of women who are managed expectantly because of chronic abruption. Bonds and colleagues [11] expectantly managed 43 women with clinical evidence of chronic abruption before 35 weeks of gestation, and achieved a mean latency period to delivery of 12.4 days. Towers and colleagues [12] also managed chronic abruption occurring between 23 and 36 weeks of gestation using a tocolytic drug, and achieved a mean latency period to delivery of 18.9 days. Intravenous tocolytic agents were administered in all of the 6 women of the CA group in this series, and the median latency to delivery was 18.5 days (3–64 days). For women with chronic abruption occurring before 34 weeks of gestation in a stable systemic condition, without maternal coagulation disorder or problems on the fetal well-being test, use of tocolytic agents with the objective of prolonging the pregnancy has been confirmed to be a reasonable treatment policy [12, 13]. However, the maternal condition developed acute abruption at a high percentage of women, both in the CA group and the PSH group, suggesting that when using tocolytic agents, strict management under informed consent from the women is necessary. Although we did not encounter any women in whom the use of tocolytics masked the symptoms of acute abruption and resulted in a delay in the detection of abruption, Oyelese and Ananth [14] have proposed that because tocolytics, especially β-stimulants, could mask the clinical signs of hemorrhage, such as tachycardia, magnesium sulfate should be selected as the first-line tocolytic agent.

Use of a tocolytic agent did not appear to increase the morbidity in either of the two groups, and the use of tocolytic agents for the management of peripheral placental hemorrhage may lead to prolongation of gestation period, suggesting the effectiveness of tocolytic agents. However, we cannot draw any definitive conclusions from this study, because of the absence of a control group. A prospective randomized trial is necessary to determine whether the use of tocolytics might be of any benefit.
